# An in-depth characterization of development-related electroretinographical and morphological changes in Landrace pigs

**DOI:** 10.1007/s10633-025-10050-1

**Published:** 2025-09-25

**Authors:** Lea Skrzypczyk, Bryan Calder Ackermann, Victor Aristide Augustin, Ulrike Rahn, Philipp Uhl, Gerd Uwe Auffarth, Maximilian Hammer

**Affiliations:** 1 David J Apple Laboratory for Vision Research and University Eye Clinic Heidelberg, Heidelberg, Germany; 2 Institute of Pharmacy and Molecular Biotechnology, Heidelberg, Germany; 3 LKC Technologies, Gaithersburg, USA; 4 Faculty of Biosciences, Heidelberg University, Im Neuenheimer Feld 400, 69120 Heidelberg, Germany

**Keywords:** Full-field electroretinography, Large animal, Pig, Optical coherence tomography

## Abstract

**Purpose:**

Landrace pigs are increasingly used as a large-animal model in ophthalmic research due to their cone-enriched visual streak and anatomical similarity to the human eye. However, they are commonly studied at 16–20 weeks of age, a timeframe in which the animals double their weight and development-related physiological changes may occur. This study aims to characterize retinal function and morphology and establish reference values for future translational studies.

**Methods:**

Landrace pigs (16–20 weeks old) underwent standardized examinations of the left eye at baseline (16 weeks), 18 and 20 weeks. The left eye was examined by optical coherence tomography (OCT), fundus photography, histology, and full-field electroretinography (ffERG) under light- and dark-adapted conditions, using the ISCEV-compliant 6-step Dog, Cat, Nonhuman Primate protocol.

**Results:**

A total of 30 animals were included. Retinal morphology remained stable throughout the study period, with no significant changes in retinal thickness observed by OCT (baseline: 252 ± 24 µm; week 20: 249 ± 11 µm; *p* = 0.17) or by histology. ffERG revealed increased amplitudes under light- and dark-adapted conditions at 20 weeks compared to baseline at 16 weeks of age (e.g. light-adapted b-wave: + 65 µV, + 18.4%, *p* < 0.01), while latencies remained stable without clinically relevant changes.

**Conclusions:**

During this phase of rapid development, Landrace pigs undergo significant functional retinal maturation without corresponding morphological changes emphasizing importance of functional testing in retinal assessments. This study provides reference data in a large number of animals.

**Supplementary Information:**

The online version contains supplementary material available at 10.1007/s10633-025-10050-1.

## Introduction

Animal models play a key role in ophthalmic research, providing valuable insights into retinal physiology, disease progression, and potential therapeutic interventions. Among these models, pigs have gained increasing attention due to their anatomical and physiological similarities to the human eye. In particular, the size of the porcine eyeball (23.9 ± 0.08 mm) closely resembles that of the human eye (23–24 mm) [1], which allows the use of standard clinical instruments and imaging modalities (such as optical coherence tomography (OCT) and fluorescein angiography), as well as intraocular surgical techniques. This is a major advantage over smaller animal models such as rodents or rabbits. Furthermore, pigs have a cone-enriched central retinal area, known as visual streak, which mimics the human macula and contains a high density of cone photoreceptors. This feature makes them particularly relevant for studies involving macular function and photoreceptor integrity. In this study Landrace pigs were used as healthy, wild type animals, allowing the functional and structural characterization of the developing retina under physiological conditions. Landrace pigs are more readily available and a cost-effective alternative to specialized breeds such as Göttingen Minipigs, making them a good choice for translational ophthalmic research.

One consideration in using Landrace pigs as an animal model is their substantial growth during experimental periods. Conventional Landrace pigs exhibit rapid growth, gaining significant body weight within months [2]. Landrace pigs are typically included in long-term experiments at around 16–20 weeks of age, as their weight can exceed 100 kg by six months, making handling increasingly difficult [3]. Indeed, fully grown Landrace pigs can reach approximately 150 kg, further potentiating these handling challenges [3]. Importantly, histomorphological and immunohistochemical studies suggest that major retinal lamination and synaptic organization are largely complete by postnatal day 28, while more subtle structural and molecular changes—such as photoreceptor outer segment elongation and increased opsin expression—continue up to approximately six months of age [4]. This developmental difference may have implications for longitudinal ophthalmic studies, particularly regarding retinal morphology and functional assessments. In practice, the period between about 16 and 20 weeks of age may be optimal for ophthalmic experiments—by this stage the retina is nearly mature, yet the animals (approximately 40–70 kg) are still of manageable size.

Several studies have investigated retinal development in swine during early postnatal life, particularly up to 3–4 months of age. For example, Fernandez de Castro et al. demonstrated functional retinal maturation from birth to P120 using full-field- electroretinography (ffERG), reporting a steep increase in rod- and cone-driven responses during this period, followed by a functional plateau thereafter**.** However, functional and structural development beyond this age remains poorly characterized, particularly in healthy wild-type Landrace pigs. This period is of particular interest, as it overlaps with a phase of rapid somatic growth, during which it remains unclear whether retinal physiology continues to mature or has already stabilized**.** At the same time, the importance of such data is emphasized by the growing number of gene therapy trials and biocompatibility studies conducted in porcine models rather than traditional rabbit models [5–7]. Recent studies have demonstrated the general feasibility of long-term functional assessments in pigs over a two-month period following such gene therapy intervention, emphasizing specifically ffERG as the most important tool to assess retinal function [6]. Similarly, recently ffERG was utilized to confirm the loss of natural retinal responses after chemically induced photoreceptor degeneration in Göttingen minipigs, ensuring that subsequent visual cortical responses originated solely from prosthetic stimulation [8]. At the same time, there is little longitudinal and normative data tracking functional changes in individual Landrace pigs over the important time frame between 16 and 20 weeks of age [9]. A comprehensive understanding of the natural variability in ffERG amplitudes and latency variations in control eyes is necessary to establish reliable baseline parameters for future experimental interventions and their interpretation.

This study aims to address this knowledge gap by analyzing changes in ffERG amplitude and latency in a large cohort of Landrace pigs over a defined experimental period. These findings help to refine our understanding of retinal function in porcine models and provide valuable information for the design of future ophthalmological studies with Landrace pigs while at the same time providing normative data in a large number of animals.

## Methods

### Animals

30 Landrace pigs, weighing between 40 and 45 kg and 16 weeks old at the start of the study, were included in this study. Pigs were acquired from a regional farmer and housed at the Interfaculty Biomedical Research Facility, Heidelberg University, in compliance with German animal welfare laws, EU directives (2010/63/EU), and ARRIVE guidelines. They had unrestricted access to water and controlled access to food. The study was approved by the local ethics committee (35-9185.81/G-11/24) and adhered to the ARVO guidelines for the use of animals in ophthalmic and vision research. The animals underwent a variety of experimental procedures on their right eye that had no effect on the left eye. Only the data of the left control eye was included in this study.

### Study protocol

**Fig. 1:**
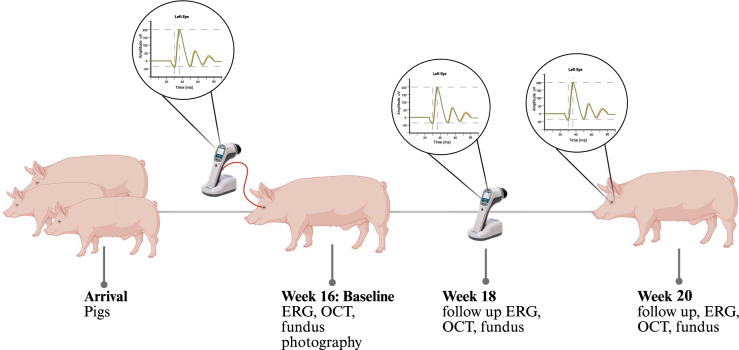
Study protocol 30 Landrace pigs underwent functional and structural retinal assessments over a four-week observation period. Baseline examinations, performed at the age of 16 weeks (one week after arrival), included full-field ERG and OCT imaging. Follow-up ERG recordings and retinal OCT were conducted at 18 and 20 weeks of age. Retinal morphology was evaluated at the same time points by optical coherence tomography (OCT), complemented by fundus photography

The baseline examination was performed when the pigs reached 16 weeks of age, approximately one week after arrival to allow animals to acclimate to the new environment. Follow-up assessments took place at 18 and 20 weeks of age (Fig. 1). For all procedures, animals were initially sedated with an intramuscular injection of Sedanol (Azaperon, WDT, Garbsen, Germany) (6 mg/kg BW). Subsequently, they were anesthetized using a combination of ketamine (CP Pharma, Burgdorf, Germany) (11 mg/kg BW) and midazolam (Hameln Pharma, Hameln, Germany) (2 mg/kg BW). Isofluran (CP Pharma, Burgdorf, Germany) was used to maintain anesthesia. Animals were euthanized after completing the final evaluation (Fig. 1).

#### IOP, fundus photography and OCT

Intraocular pressure measurements, fundus photography, ffERG and OCT were performed at each examination time point (Fig. 1). At first intraocular pressure measurements were obtained using the iCare Tonovet Plus (TONOVET Plus, iCare Finland Oy, Vantaa, Finland). Mydriasis was induced with cyclopentolate (0,5%), epinephrine (5%), and tropicamide (1%). Fundus photographs of the posterior pole, including the optic nerve head, were captured using the ClearView2 veterinary fundus camera (ClearView 2, Optibrand Ltd., Fort Collins, CO, USA). Next, optical coherence tomography (OCT) scans of the central retina were performed using the Spectralis OCT system (Spectralis OCT, Heidelberg Engineering GmbH, Heidelberg, Germany). All pigs were examined under general anesthesia while lying in lateral position. A 20° dense scan centered on the visual streak was acquired and a 30° infrared reflectance image were captured. The OCT's follow-up function ensured precise examination of the same location at any follow-up examinations.

#### Electroretinography

ERG was conducted using the RETevet™ (LKC Technologies, Gaithersburg, USA) system according to the Dog, Cat, Nonhuman Primate ISCEV 6 Step testing procedure. The protocol includes six tests designed to evaluate various aspects of retinal activity as shown in Table 1. Prior to examination full mydriasis was achieved using Cyclopentolate (Zyklolat EDO®, Bausch & Lomb, Canada), Tropicamide (Mydriaticum Stulln® DU, Pharma Stulln, Germany), Phenylephrine (Neosynephrin POS® 5%, Ursapharm Arzneimittel, Germany) and Atropine (Atropin-POS® 0,5%, Ursapharm Arzneimittel, Germany) eye drops as needed. Active ERG-Jet-Electrode (ERG-Jet™, Fabrinal SA, La Chaux-de-Fonds, Switzerland) was placed on the cornea, with Vidisic (Vidisic, Dr. Gerhard Mann chem.-pharm. Fabrik GmbH, Berlin, Germany) applied to the ocular surface to enhance adhesion. The reference needle electrode (Disposable Subdermal Needle Electrode, Natus Neurology Incorporated, Middleton, WI, USA) was positioned near the outer canthus, and the ground needle electrode (Disposable Subdermal Needle Electrode, Natus Neurology Incorporated, Middleton, WI, USA) on the forehead. Scotopic recordings were performed after a standardized dark adaptation period of 15 min.

**Table 1:** Dog, Cat, Nonhuman Primate ISCEV 6 Step ERG protocol

Test number	Name	Adaptation	Test type	Flash frequency	Flash Luminance (cd·s/m^2^)	Background illumination (cd/m^2)^
1	LA3	Light	Standard flash ERG	2 Hz	3.0	30
2	LA flicker	Light	Flicker ERG	28.3 Hz	3.0	30
3	DA0.01	Dark	Weak flash ERG	0.1 Hz	0.010	None
4	DA3	Dark	Standard flash ERG	0.1 Hz	3.0	None
5	OPs	Dark	Oscillatory potentials (ERG)	Reprocesses test 4 results with OP filter		
6	DA10	Dark	Strong flash ERG	0.05 Hz	10	None

The table outlines six ERG test conditions used under light- and dark-adapted states. Each test includes the test type, flash frequency, flash luminance (cd·s/m^2^), and background illumination (cd/m^2^)

#### Histology

At the end of the observation period, porcine eyes were removed post-mortem and fixed in 4% paraformaldehyde (ROTIHistofix 4%, Carl-Roth GmbH, Karlsruhe, Germany) for 48 h. The tissues were cryoprotected in ascending sucrose solutions (10, 20, 30) in PBS, then trimmed with a scalpel (Cryostat scalpel blade No. 24, B. Braun Melsungen AG, Melsungen, Germany) and embedded in Tissue Freezing Medium (LEICA Biosystems). Samples were frozen at −24 °C in a cryostat (LEICA CM 1850) and sectioned at 7 µm thickness. Sections were mounted on Superfrost Plus microscope slides (Epredia, Basel, Switzerland) and air-dried at room temperature for 12 h.

For H&E staining, slides were rinsed in distilled water, stained with Mayer's hematoxylin (acidic, Carl-Roth GmbH, Karlsruhe, Germany) for 6 min, blued in tap water for 15 min, rinsed again with distilled water, stained with eosin G solution (0.5%, Carl-Roth GmbH, Karlsruhe, Germany) and one drop of glacial acetic acid (99.8–100%, Bernd Kraft) for 3 min, and rinsed with tap water. The slides were dehydrated through ethanol, cleared in ROTIHistol mounted with ROTIHistokitt (both Carl-Roth GmbH, Karlsruhe, Germany), and covered with a coverslip (24 × 50 mm, borosilicate glass, VWR).

### Statistical analyses

Statistical analyses were conducted with Prism 10 (GraphPad Inc, USA) using paired t-tests, Mann–Whitney tests or paired ANOVA after ensuring normality as appropriate.

## Results

**Fig. 2:**
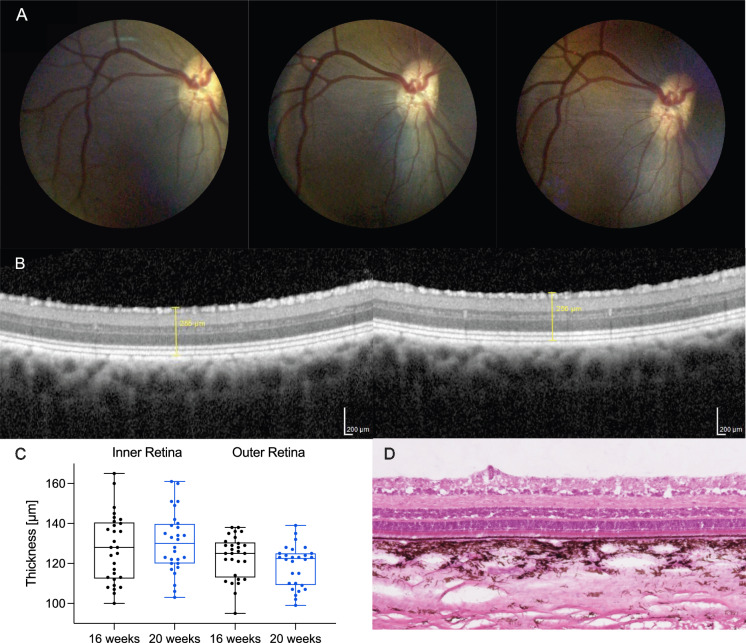
Evaluation of retinal thickness over 4 weeks using fundus photography OCT and histology **(A)** Fundus photographs taken at baseline (16 weeks of age) and after 2, and 4 weeks (up to 20 weeks of age) show an intact ocular fundus without visible pathological alterations throughout the observation period. (**B**) Representative OCT images at 16 weeks (left) and 20 weeks of age (right) show intact retinal layers and comparable retinal thickness measurements (255 µm vs. 256 µm). (**C**) Analysis of Inner and Outer retinal thickness by OCT (n = 30) over time reveals no significant changes between 16 and 20 weeks of age (*p* = 0.18 and 0.08, respectively). (**D**) Histological examination (H&E staining, 10 × magnification) confirms preserved retinal structure without morphological alterations

Retinal morphology and thickness remained stable between 16 and 20 weeks of age. All animals presented with a healthy fundus throughout the whole study period. Retinal thickness measured by OCT was not statistically significantly different at baseline (16 weeks) (252 ± 24 µm) and 4 weeks later (249 ± 11 µm), *p* = 0.17. This also held true when analyzing inner and outer retinal thickness separately. (Inner: 127 ± 17 µm vs 130 ± 15 µm, *p* = 0.18; Outer: 123 ± 11 µm vs. 119 ± 10 µm, *p* = 0.08, Fig. 2).

Histological analysis showed no damage or changes in retinal structure over time (Fig. 2). A total of 30 animals were included in the study and monitored over 4 weeks.

**Fig. 3:**
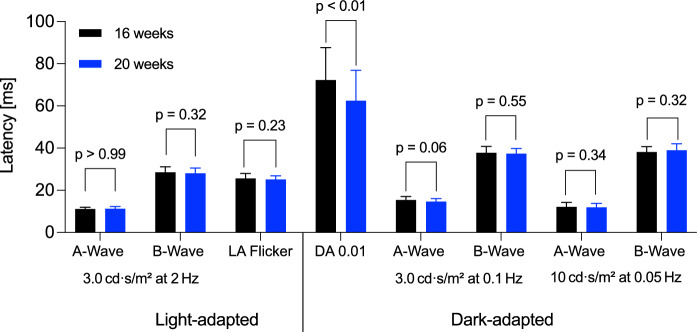
Peak latencies in Landrace pigs: Values for A- and B-waves under light- and dark-adapted conditions (n = 30) and light-adapted flicker, measured at 16 and 20 weeks of age. Data are presented as mean ± SEM, with p-values from paired t-tests

**Fig. 4:**
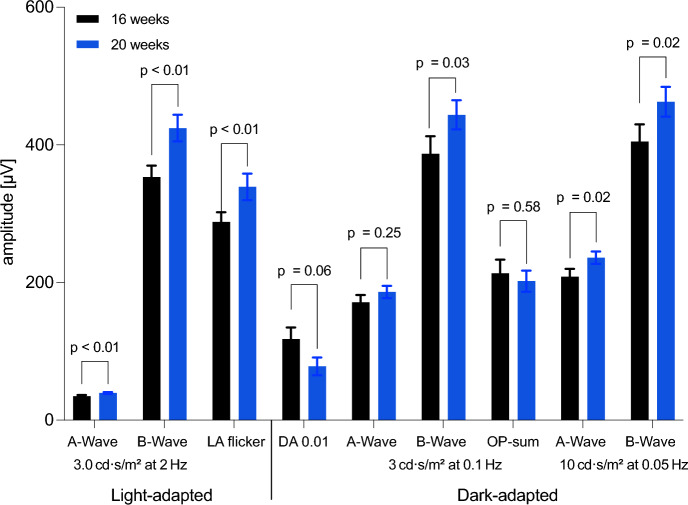
ERG amplitudes in Landrace pigs: Amplitude values of A- and B-waves as well as oscillatory potentials under light- and dark-adapted conditions (n = 30) at 16 and 20 weeks of age. Data are presented as mean ± SEM, with p-values from paired t-tests

**Fig. 5:**
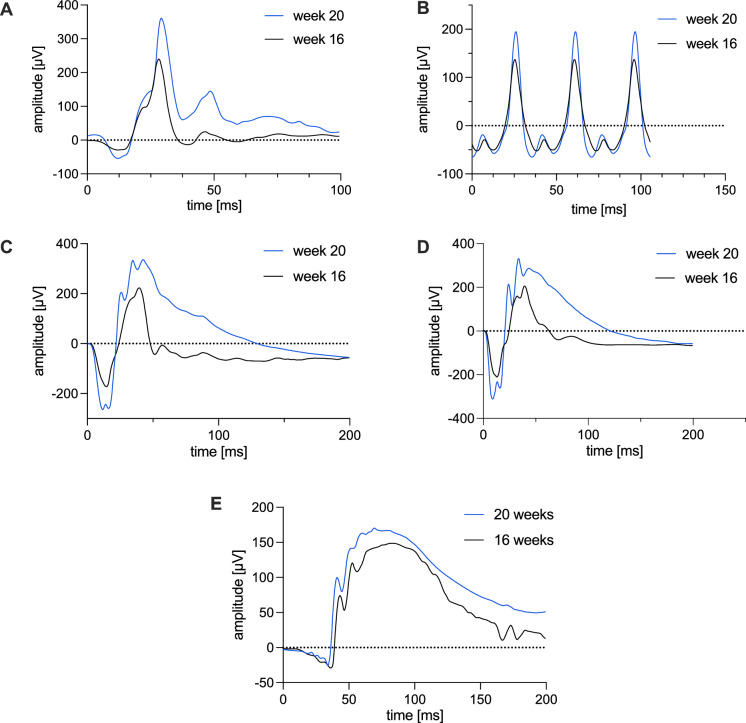
Representative ERG waveforms from an individual animal at 16 and 20 weeks of age. ERG traces from individual animals at 16 (black) and 20 weeks (blue) demonstrate greater retinal response amplitudes over time. (**A**) Light-adapted ERG waveform from the LA3 test. (**B**) LA3 flicker response (**C**) DA3 response (**D**) DA10 response (**E**) DA0.01 response

Descriptive statistics of ffERG parameters at 16 and 20 weeks of age, including mean, median, interquartile range, standard deviation, and 95% confidence intervals, are summarized in Supplementary Table S1 and serve as reference values for the observed longitudinal changes. Under light-adapted conditions, the ffERG tests showed significant increases in amplitudes compared to baseline (Figs. 4, 5). In the flash ERG (2 Hz), the a-wave amplitude increased significantly by 4.5 µV (*p* < 0.01; 95% CI: 1 to 8 µV), and the b-wave amplitude increased by 65 µV (*p* < 0.01; 95% CI: 30 to 99 µV) (Figs. 4, 5). The flicker ERG (28.3 Hz) also showed a significant increase in amplitude of 51 µV (p < 0.01; 95% CI: 16 to 86 µV) (Figs. 4, 5). Latencies did not show significant changes (a-wave: difference 0.00 ms, *p* > 0.99; b-wave: difference 0.6 ms, *p* = 0.32; LA flicker ERG: difference 0.6 ms, *p* = 0.23) (Fig. 3).

Significant increases in amplitudes were also observed under dark-adapted conditions (Figs. 4, 5). Solely, the DA 0.01 b-wave amplitude differed by −39 µV, but this change was not statistically significant (*p* = 0.06; 95% CI: –1.3 to 80.2 µV) (Fig. 4). Under DA3 conditions, only the b-wave amplitude increased significantly (+ 57 µV; *p* = 0.03; 95% CI: 6 to 108 µV), while the a-wave did not reach statistical significance (*p* = 0.25). The Oscillatory Potentials (OPs) observed during the DA3 were not statistically different between time points (*p* = 0.58; 95% CI: −29.88 to 52.66 µV). Under DA10 conditions, both a- and b-wave amplitudes increased significantly: the a-wave by + 30 µV (*p* = 0.02; 95% CI: 3 to 56 µV) and the b-wave by + 60 µV (*p* = 0.02; 95% CI: 12 to 109 µV). Latencies remained stable in most of dark-adapted tests: The DA3 a-wave latency decreased by 0.7 ms (*p* = 0.06) and the b-wave latency by 0.4 ms (*p* = 0.55), neither reached statistical significance. Similarly, DA10 latencies remained unchanged (a-wave: –0.4 ms, *p* = 0.34; b-wave: 0.7 ms, *p* = 0.32), indicating stable signal timing throughout the observation period. (Fig. 3). In contrast, DA0.01 b-wave latency decreased significantly by 10 ms (*p* < 0.01; 95% CI: –16 to –4 ms) (Fig. 3). Representative ffERG curves from one animal demonstrate the increase in amplitudes at 20 weeks of age compared to 16 weeks under both light- and dark-adapted conditions (Fig. 5).

## Discussion

In this study, we observed significant age-related changes in retinal function of Landrace pigs between 16 and 20 weeks of age. Under both light- and dark-adapted conditions, ffERG amplitudes significantly increased, especially b-wave amplitudes, while latency times and oscillatory potentials remained stable. Inner and Outer Retinal thickness measured by OCT showed no significant changes, histology showed a healthy, preserved retinal structure at the end of the experiments. These findings provide valuable natural history data for future studies using Landrace pigs in ophthalmic research.

Previous histomorphological data indicate that major lamination is complete by postnatal day 28, while more subtle remodeling—such as ONL thickening and opsin expression—continues up to approximately six months of age [4]. The observed increase in ffERG amplitudes could reflect morphological changes within the developing porcine retina. However, our OCT measurements did not show significant changes in retinal thickness or retinal substructure and morphology during the study, suggesting that ffERG amplitude increase reflect maturation in the retina caused by changes that are too small or otherwise invisible to OCT. This finding is consistent with histomorphological analyses in Göttingen minipigs and domestic pigs, which showed retinal maturation while the overall retinal thickness remains stable throughout postnatal development [4], further supporting that the increased amplitudes is rather the result of a maturation processes in retinal cells, such as photoreceptors and bipolar cells turning functionally more efficient over time [10].

Photoreceptor maturation likely plays a key role in these functional improvements [10]. Immunolabeling for recoverin and rhodopsin indicated an elongation of photoreceptor segments in six-month-old pigs compared to one-week-old animals [11]. Additionally, a histological study revealed that as photoreceptors mature, the inner segments of both rods and cones show increased synaptophysin immunolabeling, indicating a greater density of synaptic vesicles [4]. This synaptic refinement may enhance photoreceptor activity and signal transmission to bipolar cells, which could provide a mechanistic explanation for the amplitude increases observed in our cohort, although this remains speculative in the absence of direct histological or molecular correlation. The observed increase in ffERG amplitudes between 16 and 20 weeks of age suggests ongoing functional maturation. Whether this development reaches a plateau beyond this period remains unclear and warrants further longitudinal investigation. As oscillatory potentials remained mostly unchanged over the observational period, our findings suggest that retinal maturation between 16 and 20 weeks of age might not apply to amacrine or interplexiform cells, which are typically associated with oscillatory potentials [12].

In contrast to previous work by Fernandez de Castro et al., who reported that cone-driven ffERG responses plateau by postnatal day 120, our findings indicate that functional maturation may continue beyond this timepoint in healthy Landrace pigs. Similar amplitude increases have been reported in other species, such as mice and rabbits, where a standardized full-field ffERG study demonstrated that b-wave amplitudes were smaller in young rabbits (3 months old) compared to older ones (1–2 years old), indicating an increase in ffERG amplitudes with maturation [13–16]. It is unlikely that retinal ganglion cells significantly increase in number, as retinal cell populations usually decrease and stabilize after early postnatal development [17]. Histological studies in Landrace pigs showed, that the ganglion cell layer undergoes a notable reduction in cell density as the retina matures [4]. However, improved synaptic connectivity or maturation of existing cells could explain increased amplitudes.

The increase in ffERG amplitudes observed in our study may indicate enhanced retinal sensitivity and improved synaptic efficiency. Since ffERG amplitudes are widely used as functional biomarkers in preclinical and clinical research, these findings highlight the importance of understanding natural developmental changes when interpreting experimental data in porcine models [6, 8, 18]. Many authors have investigated ffERG amplitudes as indicators of retinal function in various retinal diseases and maturation, but the stability or potential changes in latency times during development have received comparatively little attention in the literature [6, 8]. Notably, Kiraly et al. also reported stable ffERG latencies in their minipig cohort, consistent with our findings, suggesting that signal transmission speed is already fully developed by three months of age in both pig breeds. Another role could be the increase in axial length of the eye in Landrace pigs [5]. This lengthening could keep the implicit time stable as the transmission pathway becomes more refined and efficient, but also longer. Alternatively, the observed stability of the latency times could indicate that the signal transmission speeds in the retina are already fully developed at the age of three months and that there is little potential for further developmental changes during our observation period and that the change of length is only of minor importance.

These findings emphasize the need for defined baseline data when evaluating retinal function in porcine models. Identifying natural developmental changes is essential for distinguishing physiological maturation from treatment effects in experimental settings. In this context, our study provides valuable insights into the natural retinal maturation of Landrace pigs and is, to our knowledge, the first longitudinal ffERG analysis in this breed. Analyzing the ffERG responses in control eyes, the observed changes reflect natural development rather than experimental interventions, establishing a reliable baseline for future ophthalmic research. OCT measurements confirmed that while functional maturation occurs, retinal thickness remains stable. Further, to improve comparability we used a highly standardized, multi-step ffERG protocol from the International Society for Clinical Electrophysiology of Vision.

However, some limitations should be considered. While OCT was utilized to assess retinal thickness, histological examinations were only performed at 6 weeks. Due to ethical considerations (3R-principle), early histological analysis was not feasible. Furthermore, including axial length measurements could have aided in interpreting the observed difference since a correlation between axial length and ERG-response has been described in the literature [19–22]. In addition, the duration of the study was limited to 6 weeks due to the rapid growth of the pigs and the associated increasing challenges in handling larger animals, as well as the timeline of additional experiments performed on partner eyes. This relatively short timeframe may not capture the full extent of retinal maturation in Landrace pigs. A longer study period could potentially reveal additional developmental changes. Also, other pig breeds, such as the increasingly utilized Göttingen Mini Pig and female Landrace pigs should be evaluated in further studies [5].

## Conclusion

In conclusion, the significant increase in amplitude observed in our study may be related to structural maturation and synaptic refinement rather than changes in retinal cell numbers. These data establish an important reference for interpreting functional changes in Landrace pigs in future ophthalmological research.

## Supplementary Information

Below is the link to the electronic supplementary material.

Supplementary file1 (DOCX 19 KB)
