# Supplementary material for: An in-depth characterization of development-related electroretinographical and morphological changes in Landrace pigs
Source: Doc Ophthalmol. 2025 Sep 25;151(3):219–28. doi: 10.1007/s10633-025-10050-1 (PMC12568845; doi:10.1007/s10633-025-10050-1)
Supplement: Supplementary file 1 — Supplementary file1 (DOCX 19 KB) [file 10633_2025_10050_MOESM1_ESM.docx]

**Supplementary Table S1.** Descriptive statistics of the recorded parameters at 16 and 20 weeks of age. Reported are the mean, median, interquartile range (IQR), standard deviation (SD), and the 95% confidence interval (CI) of the means.

|  | parameter | age | mean | median | IQR | SD | 95%CI |
| --- | --- | --- | --- | --- | --- | --- | --- |
| LA3  a-wave | amplitude | 16w | 35.13 | 34.35 | 30.00 to 39.55 | 8.54 | 31.94 to 38.32 |
|  | amplitude | 20w | 39.86 | 40.65 | 33.68 to 45.35 | 8.20 | 36.80 to 42.93 |
|  | latency | 16w | 11.10 | 11.20 | 10.40 to 11.55 | 0.90 | 10.75 to 11.45 |
|  | latency | 20w | 11.10 | 11.00 | 10.30 to 11.55 | 1.05 | 10.70 to 11.50 |
| LA3  b-wave | amplitude | 16w | 354.2 | 345.5 | 281.5 to 418.8 | 89.27 | 320.9 to 387.6 |
|  | amplitude | 20w | 419.2 | 401.0 | 327.3 to 516.8 | 112.4 | 377.2 to 461.1 |
|  | latency | 16w | 28.65 | 28.60 | 26.50 to 31.00 | 2.59 | 27.69 to 29.60 |
|  | latency | 20w | 28.06 | 28.05 | 26.10 to 29.75 | 2.56 | 27.10 to 29.02 |
| LA0.01  b-wave | amplitude | 16w | 287.9 | 282.0 | 226.8 to 352.3 | 76.70 | 259.3 to 316.5 |
|  | amplitude | 20w | 338.9 | 307.0 | 245.5 to 416.0 | 109.7 | 297.9 to 379.8 |
|  | latency | 16w | 25.63 | 25.45 | 24.05 to 26.68 | 2.32 | 24.77 to 26.50 |
|  | latency | 20w | 25.02 | 25.00 | 23.50 to 25.78 | 1.76 | 24.36 to 25.67 |
| DA 0.01  b-wave | amplitude | 16w | 117.8 | 116.5 | 24.23 to 193.3 | 92.68 | 83.16 to 152.4 |
|  | amplitude | 20w | 78.32 | 44.55 | 21.80 to 137.0 | 70.32 | 52.05 to 104.6 |
|  | latency | 16w | 72.31 | 73.30 | 64.53 to 84.13 | 14.31 | 66.60 to 78.03 |
|  | latency | 20w | 62.48 | 61.55 | 49.68 to 73.73 | 14.37 | 57.12 to 67.85 |
| DA3  a-wave | amplitude | 16w | 172.0 | 173.0 | 123.5 to 223.5 | 58.71 | 150.1 to 194.0 |
|  | amplitude | 20w | 185.8 | 187.0 | 145.5 to 187.0 | 47.98 | 167.9 to 203.7 |
|  | latency | 16w | 15.39 | 15.00 | 14.10 to 17.13 | 1.77 | 14.73 to 16.05 |
|  | latency | 20w | 14.63 | 14.50 | 13.88 to 15.70 | 1.38 | 14.12 to 15.14 |
| DA3  b-wave | amplitude | 16w | 389.1 | 403.0 | 303.5 to 496.5 | 141.0 | 336.5 to 441.8 |
|  | amplitude | 20w | 446.5 | 444.0 | 393.5 to 516.8 | 112.7 | 404.4 to 488.6 |
|  | latency | 16w | 37.77 | 38.00 | 35.30 to 40.60 | 2.97 | 36.68 to 38.86 |
|  | latency | 20w | 37.39 | 37.40 | 35.53 to 39.23 | 2.62 | 36.41 to 38.37 |
| DA10  a-wave | amplitude | 16w | 209.6 | 200.0 | 156.8 to 260.0 | 62.22 | 186.4 to 232.9 |
|  | amplitude | 20w | 239.2 | 243.5 | 202.5 to 271.5 | 50.75 | 220.2 to 258.1 |
|  | latency | 16w | 12.23 | 12.15 | 10.20 to 13.38 | 2.27 | 11.38 to 13.08 |
|  | latency | 20w | 11.85 | 11.45 | 10.30 to 13.30 | 1.89 | 11.14 to 12.56 |
| DA10  b-wave | amplitude | 16w | 406.8 | 426.0 | 297.0 to 516.5 | 138.1 | 355.2 to 458.4 |
|  | amplitude | 20w | 467.1 | 477.0 | 409.3 to 536.0 | 119.3 | 422.5to 511.7 |
|  | latency | 16w | 37.76 | 37.70 | 35.45 to 40.30 | 2.79 | 36.72 to 38.80 |
|  | latency | 20w | 38.49 | 37.80 | 36.48 to 40.23 | 3.61 | 37.15 to 39.84 |
